# The Evolving Importance of Insulin Signaling in Podocyte Health and Disease

**DOI:** 10.3389/fendo.2018.00693

**Published:** 2018-11-21

**Authors:** Abigail C. Lay, Richard J. M. Coward

**Affiliations:** Bristol Renal, Bristol Medical School, Faculty of Health Sciences, University of Bristol, Bristol, United Kingdom

**Keywords:** podocyte, insulin signaling, diabetic kidney disease (DKD), insulin resisitance, diabetes, albuminuria, podocyte metabolism

## Abstract

Diabetic kidney disease (DKD) is the leading cause of end-stage renal disease worldwide, occuring in approximately one-third of diabetic patients. One of the earliest hallmarks of DKD is albuminuria, often occurring following disruptions to the glomerular filtration barrier. Podocytes are highly specialized cells with a central role in filtration barrier maintenance; hence, podocyte dysfunction is a major cause of albuminuria in many settings, including DKD. Numerous studies over the last decade have highlighted the importance of intact podocyte insulin responses in the maintenance of podocyte function. This review summarizes our current perspectives on podocyte insulin signaling, highlighting evidence to support the notion that dysregulated podocyte insulin responses contribute toward podocyte damage, particularly during the pathogenesis of DKD.

## Introduction

Insulin is a metabolic hormone, essential in regulating systemic glucose levels and whole-body metabolism. While the primary function of insulin is to enhance glucose uptake into classically insulin-responsive tissues, including skeletal muscle, adipose tissue, and liver; several other cellular responses are also directly regulated by insulin, including fatty acid synthesis, growth, apoptosis, transcription, and translation (1, 2). In this regard, insulin can directly influence a range of cells and tissues, contributing to systemic homeostasis. In the kidney, insulin acts at multiple sites along the nephron, including in the glomerulus (3–5) and throughout the renal tubule (6–8).

Insulin resistance is a common metabolic abnormality which plays a central role in the pathogenesis of both type 1 and type 2 diabetes. Insulin resistance is also linked to renal injury, including the development of albuminuria and DKD (9–15). While numerous circulating factors are dysregulated in conditions of systemic insulin resistance, including various nutrients, metabolites, and proinflammatory cytokines, it is increasingly well-recognized that the disruption of metabolic pathways in intrinsic renal cells, including insulin signaling pathways, are key drivers of kidney damage.

Podocytes sit on the urinary side of the glomerular filtration barrier and have a critical role in glomerular function. They are highly-specialized, terminally-differentiated cells with a limited capacity for renewal, thus relying on their ability to sense and adapt to environmental changes and stimuli. Podocyte loss occurs early in many albuminuric conditions and is one of the earliest features observed in diabetic kidney disease (DKD) (16–21). Over the last decade, many studies have highlighted the importance of podocyte insulin signaling in the maintenance of glomerular function; this review summarizes our current perspectives on podocyte insulin responses, highlighting recent advances in this field and focusing on the notion that dysregulated podocyte insulin signaling occurs in, and contributes toward the pathogenesis of, albuminuria and DKD.

## An overview of podocyte insulin signaling

The insulin signaling cascade is a complex intracellular network, involving multiple points of regulation, divergence and interaction with other signaling pathways. Briefly, insulin binding to the insulin receptor (IR; of which there are two isoforms, IR-A and IR-B, which have both structural and functional differences) stimulates IR kinase activity, autophosphorylation and the phosphorylation of insulin receptor substrate (IRS) proteins which subsequently act as docking sites, recruiting Src homology 2 (SH2)-domain-containing proteins to facilitate downstream signal transduction. These SH2-domain-containing proteins include the p85 subunit of PI3K and Grb2, thereby mediating the activation of PI3K/Akt and MEK/MAPK signaling cascades, respectively (1, 2). Importantly, while the majority of cells express IRs and several components of the signaling cascade, the downstream actions of insulin-signaling are largely dependent on cell-type. The relative expression of key signal transducers and their isoforms (including IR-A/-B, IRS1-4, and Akt1-3), expression of suppressors or enhancers, activity of interacting signaling networks, in combination with duration (and level) of insulin stimulus all contribute toward the cellular- (and context-) specificity of insulin action (1, 2).

**Figure 1:**
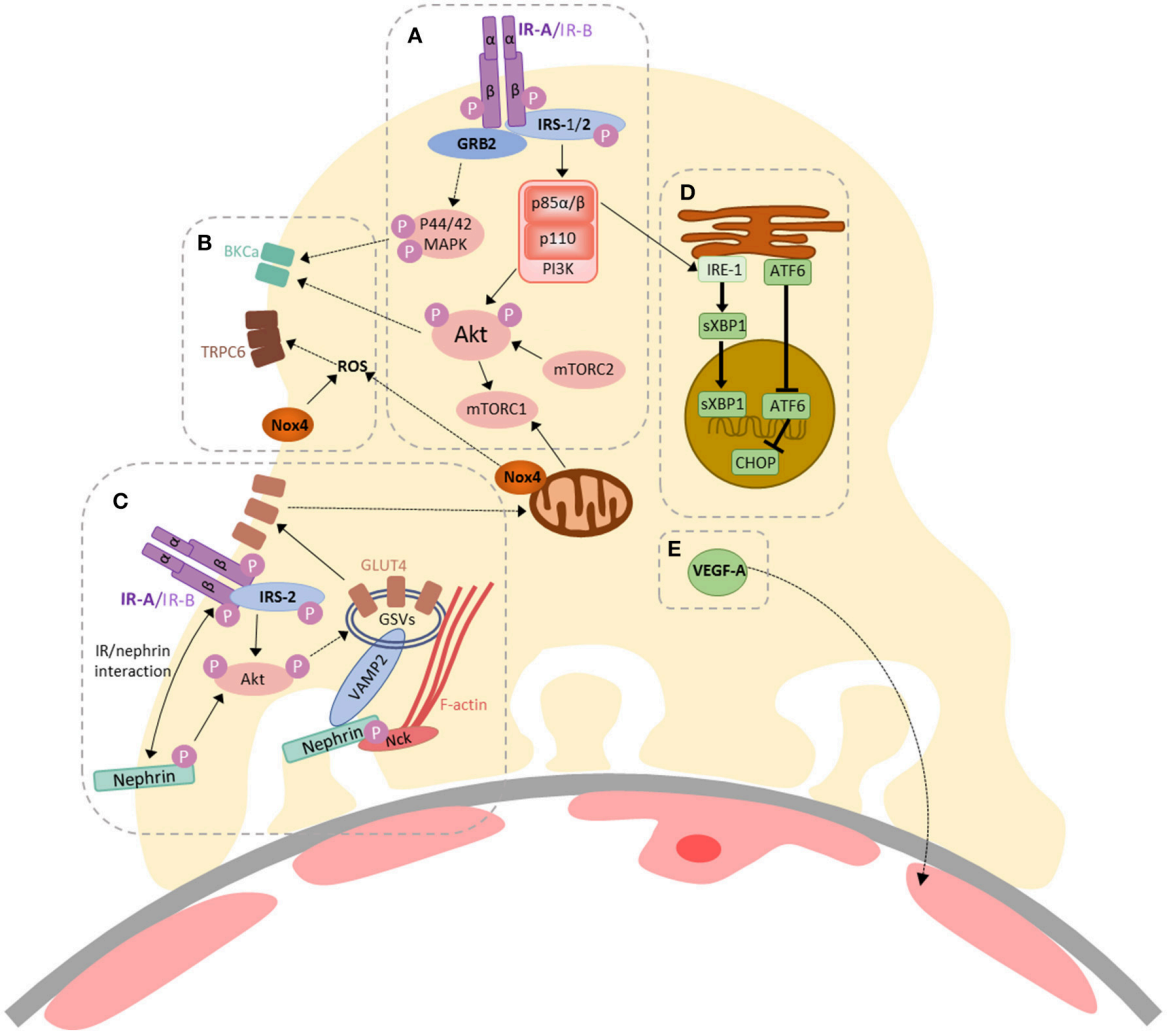
A summary of insulin-stimulated signaling in podocytes. **(A)** Activation of the insulin receptor (IR) (-A or -B isoforms) triggers auto-phosphorylation, facilitating binding and tyrosine phosphorylation of the insulin receptor substrate (IRS) proteins 1 and 2, which act as scaffolds for downstream signaling events. GRB2 is an example of an SH2-domain-containing protein, responsible for facilitating activation of Ras–MAPK signaling, resulting in p44/42 MAPK phosphorylation. Phosphoinositide 3-kinase (PI3K) is recruited via the p85 regulatory subunit, ultimately resulting in Akt phosphorylation (at Thr308). The mammalian target of rapamycin complex 2 (mTORC2) is responsible for Akt phosphorylation at Ser473. Akt can also activate mTORC1; **(B)** Insulin-stimulated contractility is regulated by calcium mobilization, via co-ordinated action of BK channels and TRPC6, which are regulated by Akt/p44/42 MAPK signaling (29) and increased ROS production (28), respectively. Insulin-stimulated dimerization of PKGIα, which may also involve TRPC6 (30), also contributes toward podocyte contractility (27); **(C)** Insulin-stimulated glucose-uptake in podocytes (4) is dependent on the expression and function of IRS-2 (31), Akt and nephrin (26, 32). Signaling via PI3K/Akt pathways can promote the translocation of GLUT4-storage vesicles (GSVs) to the plasma membrane. Nephrin plays a role in the docking and fusion of GSVs at the plasma membrane and F-actin re-organization (26, 32); **(D)** Insulin-signaling via p85α/β is involved in the adaptive ER stress response in podocytes; promoting the nuclear localization of sXBP1. Loss of podocyte IR signaling promotes increases in nuclear ATF6 and CHOP expression (24); **(E)** Podocyte VEGF-A expression is also modulated by insulin (25).

In 2005, our group found that podocytes were insulin-sensitive cells, able to rapidly increase cellular uptake of glucose, via GLUT1 and GLUT4 glucose transporters, following insulin stimulation (4). The importance of intact podocyte insulin responses for glomerular function was subsequently highlighted in podocyte-specific IR knock-out mice, which develop features of DKD, despite normal blood glucose levels (3). Insulin has since been shown to modulate several downstream responses in podocytes including changes in mitochondrial function (22), autophagy (23), ER stress (24), VEGF-A secretion (25), actin dynamics (26), contractility (27), albumin permeability (27–29), and calcium mobilization (28, 30). Current knowledge of insulin-stimulated responses in podocytes is summarized in Figure 1 (2, 33).

Of note, when compared to the other glomerular cells in primary culture, podocytes have the highest level of IR expression (5), indicating the importance of intact insulin responses in these cells. Both the IR-A and IR-B isoforms are expressed in podocytes, although IR-A is more abundant at the mRNA level (26). It is important to note here the difficulty in studying these IR subtypes, largely due to the isoforms differing in only 12 amino acids at the α-subunit carboxyl terminus (34), and whether the relative expression of IR-A or IR-B has any implications for the activation of selective signaling cascades or downstream responses in podocytes is not currently known. Following podocyte IR activation, both IRS-1 and IRS-2 are phosphorylated, which are likely to have distinct yet overlapping functions. It has been suggested that IRS-2 is the predominant isoform mediating insulin-stimulated PI3K activation in podocytes, including Akt activation and glucose uptake, due to the finding that IRS-1 cannot fully compensate for the loss of IRS-2 (31).

## Akt and mTOR

One of the central signaling molecules activated following insulin stimulation is Akt, of which there are three isoforms, Akt1-3. Of these isoforms, it is the disruption of Akt2 that is associated with impaired glucose uptake, insulin resistance and diabetes in humans and mice (35, 36), indicating that Akt2 is particularly important in mediating metabolic insulin responses. Importantly, Akt2 is also critical for podocyte survival in models of CKD, specifically in conditions of nephron-reduction (37). Of the glomerular cells, Akt2 is predominantly expressed in both mouse and human podocytes, where it is activated in situations of glomerular stress and CKD. This activation of Akt2 is considered to protect against the development of renal injury, as a podocyte-specific deletion of Akt2 has been shown to result in a more rapid disease progression (37).

These studies further suggested that the proteinuric effects of rapamycin observed in renal transplant patients with severe nephron reduction may be attributed to the inhibition of Akt2 (37), highlighting the links between the activity of Akt2 and mTOR in podocytes. In other cell systems, it is well-established that mTORC2 is responsible for Akt phosphorylation at Ser473 (38, 39), mTORC1 activity occurs downstream of Akt (39) and, notably, the chronic inhibition of mTORC2 with rapamycin promotes insulin-resistance in several model organisms (40, 41). In podocytes, signaling via the IR has been shown to influence mTOR activity (22) and, importantly, while regulated mTOR activity is essential for podocyte function, podocyte-specific over-activation of mTORC1 induces podocyte injury and plays an important role in DKD development (42, 43). Prolonged activation of mTORC1 has also been linked to insulin-resistance (44), with rapamycin treatment reversing insulin-resistance in these settings (39).

## The role of nephrin in insulin signaling

Nephrin is a podocyte-specific protein in the kidney, essential for podocyte function (45, 46). The necessity of nephrin in the maintenance of filtration barrier integrity is highlighted by the numerous nephrin mutations which cause severe nephrotic syndrome (47, 48). The importance of this protein in podocyte insulin signaling was first demonstrated in 2007, with the finding that nephrin expression was essential for podocyte GLUT1 and GLUT4 trafficking (32), which may be dependent on the interaction of nephrin with VAMP2 (32) and the requirement of nephrin for efficient insulin-stimulated actin remodeling (26). Furthermore, factors linked to the development of podocyte insulin-resistance also have effects on nephrin function and phosphorylation (49).

The importance of nephrin in regulating insulin action has been further implied by studies in other cell types. Although nephrin is a podocyte-specific protein in the kidney, there are a handful of sites around the body where nephrin is also expressed, including in pancreatic beta-cells. The importance of nephrin in controlling beta-cell insulin sensitivity has been demonstrated recently, as both patients with nephrin mutations and mice with a beta-cell-specific nephrin deletion have a reduced glucose tolerance, likely due to impaired insulin secretion (50).

Interestingly, nephrin can also directly interact with the IR (specifically the IR-B isoform) in podocytes and in glomeruli (50), which may impact on IR function. This interaction was shown to promote the selective activation of insulin signaling pathways; inhibiting the insulin-stimulated p70S6K activation, while the insulin-stimulated activation of Akt was unaffected. It would be interesting to further determine whether the selective activation of Akt isoforms also occurs (and is potentially responsible) in these settings. It is also of note that nephrin can activate PI3K/Akt pathways (51), including p70S6K phosphorylation, independent of insulin signaling (50). As nephrin dysregulation also occurs early in DKD, further exploration of the role of the role of nephrin in podocyte insulin signaling is likely to be beneficial.

## The role of podocyte insulin resistance in disease

Insulin resistance is a major metabolic abnormality with a central role in the pathogenesis of both type 1 and type 2 diabetes, including the development of renal damage (10–12, 14, 52); even in non-diabetic individuals, insulin resistance is associated with the development of albuminuria (52). This association between insulin resistance and renal disease has been further highlighted recently in a study by Ahlqvist et al., which re-classified 4 independent cohorts of diabetic patients into 5 novel clusters based on several characteristics, including the level of systemic insulin resistance. Interestingly, the patient cluster that was defined as being most insulin resistant had the highest risk of developing DKD (9).

The systemic insulin-resistant environment is associated with a dysregulation of several circulating metabolites including free fatty acids, glucose, insulin, and inflammatory cytokines, all of which have been shown to influence podocyte function in DKD. The inflammatory cytokine TNF, for example, is linked to the development of insulin resistance (53), associated with DKD progression (along with the receptors TNFR1 and TNFR2) and directly causes podocyte injury (54).

It is important to note, however, that the development of cellular insulin-resistance is often tissue-specific, and several factors associated with systemic insulin resistance may in fact enhance signaling in cells otherwise un-responsive to physiological insulin levels (55). In addition, branches of insulin-stimulated signaling pathways may be selectively impaired within cells (56, 57).

The first indication that podocytes become insulin-resistant in a diabetic environment was in findings from type-2 diabetic mice; podocytes isolated from *db/db* animals had a reduction in insulin-stimulated Akt phosphorylation, which was associated with increased apoptosis (58). In the following years, several circulating factors associated with systemic insulin resistance were shown to directly disrupt podocyte insulin signaling via several cellular mechanisms, as recently reviewed (2). We have since demonstrated that hyper-stimulation of podocytes with insulin also causes insulin-resistance, by promoting IR degradation, although nephrin expression is also required for selective downstream responses; including glucose uptake and reorganization of filamentous actin (26).

**Figure 2:**
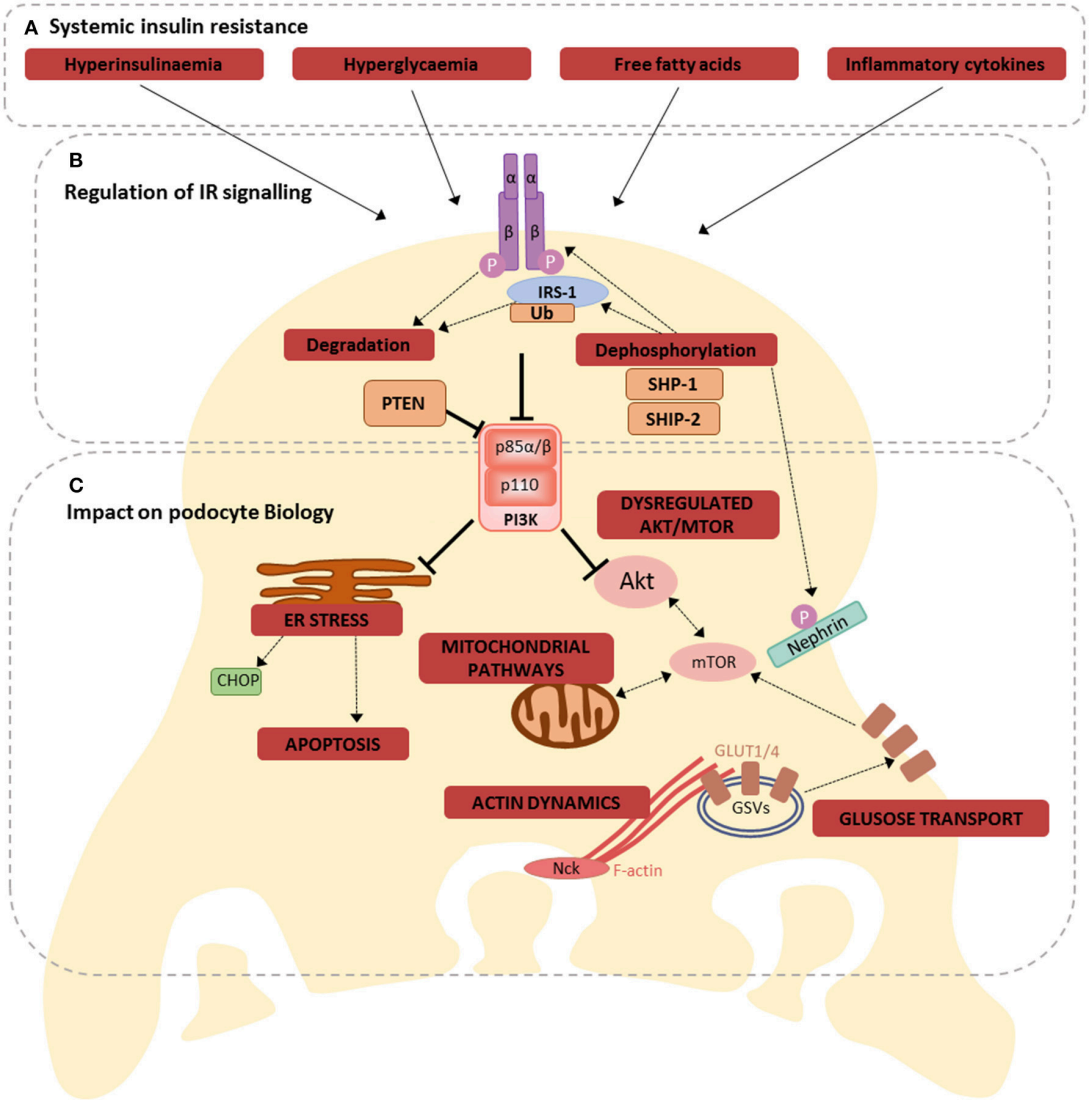
Consequences of losing dynamic IR signaling in podocytes. **(A)** Hyperinsulinaemia, hyperglycaemia, free fatty acids, and inflammation are all factors associated with systemic insulin resistance, that have been shown to disrupt podocyte insulin signaling, via several different mechanisms (60); **(B)** these mechanisms include directly affecting IR expression, increased ubiquitination of IRS-1, regulation of several proteins involved in insulin signaling, including SHP-1, SHIP-2, PTEN, ultimately disrupting downstream signal transduction; **(C)** Dysregulated IR signaling (either loss of IR signaling or uncontrolled activation of selective signaling branches) has the potential to influence several inter-connected metabolic pathways in podocytes. These include ER stress responses (promoting apoptosis), mitochondrial signaling, Akt and mTOR signaling, glucose transport and the regulation of F-actin dynamics.

As mentioned, the importance of intact podocyte insulin responses is highlighted in studies of podocyte-specific IR-knock-out (PodIRKO) mice, which develop a glomerular phenotype with features reminiscent of DKD, including albuminuria, despite maintaining normal blood glucose levels (3). In addition, in models of type 1 DKD a podocyte-specific haploinsufficiency of the IR causes a worsened phenotype, including exacerbated albuminuria (24), further highlighting the importance of intact podocyte insulin responses in disease. It is therefore reasonable to deduce that the disruptions to podocyte insulin signaling, occurring in conditions of diabetes and systemic insulin resistance (26, 58), directly contribute toward disease, particularly in the early stages of DKD development (2, 59). A summary of the implications of insulin resistance for podocyte biology is presented in Figure 2.

## Epigenetic control of podocyte insulin signaling

Epigenetic mechanisms are heritable changes in gene expression that are not a consequence of changes to primary DNA sequences. Many environmental factors influence the epigenome, including several metabolites associated with systemic insulin resistance. Importantly, epigenetic alterations may persist long after the removal of the initial insult and, as such, are often used to explain the concept of “metabolic memory”; a phenomenon whereby a transient disruption in metabolites confers long-term changes in cells and tissues. Several epigenetic mechanisms have been linked to the development of DKD (61, 62). One of the first studies to explore the relationship between changes in podocyte metabolism and the epigenome found that hyperglycaemia can induce H3 acetylation in the p66Shc promoter region, enhancing p66Shc expression, which is in turn associated with increased ROS generation (63).

More recently, the role of epigenetic modifications in controlling podocyte insulin sensitivity has been investigated. Specifically, the circulating fatty acid palmitate, known to cause podocyte insulin-resistance (2, 64), has been shown to induce a “metabolic memory” effect in podocytes, with palmitate-induced insulin-resistance persisting long after palmitate removal (65). This study demonstrated that this persistent insulin-resistance was associated with global decreases in histone H3K27 tri-methylation and increases in histone H3K36 di-methylation within the FOXO1 promoter region (65); thereby indicating that epigenetic mechanisms may be responsible for the long-lasting effects of palmitate in podocytes.

Similarly, persistent insulin-resistance associated with increased SHP-1 expression has been shown via a “hyperglycaemic memory” effect in podocytes (59). While elevations in podocyte SHP-1 had previously been linked to podocyte insulin resistance following high glucose exposure (66), this more recent study found elevations in SHP-1 expression were sustained after the normalization of glucose levels in both diabetic glomeruli and in podocytes. This sustained SHP-1 expression was associated with epigenetic changes to the promoter region of SHP-1 (specifically histone H3K4me1 and H3K9/14ac) (59).

Although the understanding of the cell-specific nature of epigenetic modifications DKD is still in its infancy, these studies indicate that changes in the podocyte epigenome may be responsible for persistent podocyte insulin-resistance in diabetes. Further research will no doubt reveal additional cell-specific epigenetic changes in DKD and their relative contribution to podocyte dysfunction.

## Insulin and podocyte ER stress

The endoplasmic reticulum (ER) plays an important role in cell maintenance. During ER stress, the unfolded protein response (UPR) is triggered as an adaptive response, to restore ER homeostasis, limiting protein synthesis, correcting protein folding, and/or promoting the degradation of misfolded proteins. However, prolonged or unresolved ER stress results in the UPR pathways triggering apoptosis (67). Several studies have demonstrated that ER stress is involved in DKD progression and, in podocytes, several factors associated with systemic insulin resistance can induce ER stress, including hyperglycaemia and free fatty acids (68, 69). Notably, in other cell types, ER stress can also inhibit IR signaling (69), although whether ER stress also contributes toward podocyte insulin-resistance is unknown.

Recently, the importance of insulin signaling in the regulation of podocyte ER stress responses has been highlighted. Insulin signaling, through p85α and p85β subunits of PI3K, has been shown to control podocyte ER stress responses, by promoting the nuclear localization of sXBP1 (24). Studies using podocyte-specific heterozygous IRKO mice *in vivo* demonstrated that a reduction in podocyte IR signaling reduced the nuclear translocation of sXBP1 and concurrent increases in nuclear ATF6 and CHOP expression which was, importantly, associated with a heightened DKD phenotype (24). Similar phenotypes were also observed in both podocyte-specific p85α KO and whole-body p85β KO mice, indicating the importance of signaling via IR-PI3K (specifically p85α/β) in mediating sXBP1 activation and adaptive ER stress responses in DKD.

Recent work from our own group has expanded on these studies, further exploring the role of podocyte insulin sensitivity and resistance in maladaptive ER stress *in vitro*. We found that enhancing podocytes insulin sensitivity in two independent cell models; stable IR overexpression and stable knock-down of PTP1B; was able to protect against several ER stress responses including increased CHOP expression and apoptosis (70). Conversely, a stable knock-down of PTEN in podocytes (which results in PI3K over-activation, in both stimulated and unstimulated cells) resulted in an increase of the ER stress response and apoptosis. This particularly highlights the importance, if not necessity, of regulated activation of these signaling pathways. This work also implies that the protective effects of IR signaling in maladaptive ER stress is not solely mediated by PI3K/Akt activity in isolation.

## Insulin signaling and podocyte mitochondrial function in DKD

Mitochondria, and mitochondrial signaling, represents another network essential in the regulation of cellular metabolism and homeostasis. Mitochondrial dysfunction is recognized as another primary event occurring in the pathogenesis of DKD, resulting in the increased generation of reactive oxygen species (ROS), including superoxide (13). In podocytes, increased ROS production in diabetes (from both the plasma membrane and mitochondria) promotes apoptosis and cell loss (71). Given the importance of mitochondria in the regulation of cellular metabolism and metabolic pathways, it is unsurprising that podocyte insulin signaling has also been linked to mitochondrial pathways (22). Insulin has been shown to enhance Nox4-dependent ROS production (23, 27), a ubiquitous NAD(P)H oxidase which is present in mitochondria (72). Nox4 has also been implicated in insulin-regulated autophagosome maturation (23), although whether this is a consequence of mitochondrial-specific ROS production is to be determined.

Mitochondrial signaling pathways also have complex and important roles aside from respiration and reactive oxygen species generation. The podocyte-specific knock-out of Phb2 (a protein indispensable for mitochondrial fusion and integrity) causes albuminuria and a severe kidney phenotype, despite mitochondrial respiration and ROS generation remaining intact. In fact, the phenotype was linked to the hyperactivation of mTOR signaling and both treatment with rapamycin and specifically reducing IR/IGF-IR signaling limited mTOR hyper-activity, ameliorating renal damage, and prolonging survival (22). This study not only explicitly links podocyte insulin signaling with mitochondria, but also indicates that over-activation of certain branches of podocyte insulin signaling pathways can be detrimental, again demonstrating the importance of regulated IR activity. Further investigation of the relationship between podocyte mitochondria and insulin sensitivity is likely to be beneficial in further understanding podocyte metabolism, particularly in DKD.

## The relationship between insulin and glucose transport in podocytes

Enhanced glucose uptake is one of the classical insulin-sensitive responses. Podocytes express many glucose transporters, including the insulin-sensitive transporters GLUT1 and GLUT4, and rapidly uptake glucose in response to insulin (4, 73, 74). Importantly, both the expression of glucose transporters and insulin-sensitive glucose uptake are dysregulated in podocytes in DKD (26, 64, 75). The relationship between podocyte glucose transporters and the development of DKD has been recently reviewed (76), so will not be covered in detail, but it is important to discuss the seemingly conflicting results from podocyte-specific GLUT1-over-expressing (77) and GLUT4-knock-out (75) mice, as both of these models were found to protect against the development of DKD. These apparent inconsistencies may be attributed to the differential regulation of GLUT1 and GLUT4 expression that seems to occur in DKD; indicating a divergence in the pathways regulating GLUT1 and GLUT4.

While both GLUT1 and GLUT4 are insulin-sensitive glucose transporters, their activity is also regulated by other signaling molecules independent of the IR, such as AMPK (78), and the protective effects of GLUT4-deficiency observed were likely independent of the IR. This again highlights the complexity of IR signaling networks and interaction with other signaling cascades. It also provides another example whereby a loss of selective branches of insulin signaling may in fact be beneficial in certain settings; in this case again the over-activation of mTOR.

## Therapeutic potential of protecting podocyte insulin signaling

The role of podocyte injury in the development of albuminuria is well-established, occurring early in the pathogenesis of many albuminuric conditions, including DKD. Thus, strategies to prevent podocyte damage and albuminuria are attractive therapeutic options in the treatment of many forms of chronic kidney disease. Given the collective evidence demonstrating that podocyte insulin signaling is disrupted in disease, and that the disruption to podocyte insulin responses (either loss of IR signaling or uncontrolled activation of selective signaling branches) is detrimental, it stands to reason that that strategies to protect podocyte insulin signaling may be beneficial in the treatment of albuminuric renal disease; particularly in the setting of systemic insulin resistance.

Supporting this notion, the insulin-sensitizing drug rosiglitazone can have direct, protective, effects on podocyte insulin responses (74) and similar, systemic, insulin-sensitizing drugs can protect against albuminuria, in both experimental diabetic nephropathy models and clinical studies (79–81), suggesting that this beneficial effect may be in part mediated by protecting podocyte insulin signaling.

Furthermore, our group have recently demonstrated that enhancing IR expression protects against ER-stress-mediated apoptosis in podocytes (70). Importantly, however, reducing PTEN expression (resulting in a consistent over-activation of PI3K signaling) has a negative effect on ER stress responses, again highlighting the importance of regulated, dynamic IR signaling.

## Summary

The importance of podocyte insulin signaling in glomerular function has been highlighted in several studies over the last decade. It is also becoming increasingly well-recognized that podocyte insulin responses are dysregulated in conditions of systemic metabolic dysfunction, including diabetes, contributing towards albuminuria in these settings. Coupled with the knowledge that several other essential metabolic pathways interact with insulin-stimulated networks in podocytes, this makes IR signaling an attractive target for therapeutic intervention. Future work in this area will no doubt advance our understanding of these signaling cascades and highlight the potential of podocyte IR-signaling as an early intervention in DKD.

## Author contributions

AL and RC conceived and wrote the manuscript.

### Conflict of interest statement

The authors declare that the research was conducted in the absence of any commercial or financial relationships that could be construed as a potential conflict of interest.
